# (*E*)-*N*′-(3,4-Dihydroxy­benzyl­idene)-4-nitro­benzohydrazide

**DOI:** 10.1107/S1600536809029705

**Published:** 2009-07-31

**Authors:** Feng Qiu, Xiao-Jing He, Ya-Xin Sun, Xu Zhu

**Affiliations:** a Shengjing Hospital of China Medical University, Shenyang 110004, People’s Republic of China

## Abstract

In the title Schiff base compound, C_14_H_11_N_3_O_5_, the dihedral angle between the two benzene rings is 1.6 (1)°. The mol­ecule displays an *E* configuration about the C=N bond. An intra­molecular O—H⋯O hydrogen bond is observed. In the crystal, mol­ecules are linked into layers parallel to (101) by O—H⋯O, N—H⋯O and C—H⋯O hydrogen bonds. One of the hydroxyl groups is disordered over two positions, with occupancies of 0.643 (5) and 0.357 (5).

## Related literature

For the biological properties of Schiff base compounds, see: Kucukguzel *et al.* (2006 ▶); Khattab (2005 ▶); Karthikeyan *et al.* (2006 ▶); Okabe *et al.* (1993 ▶). For bond-length data, see: Allen *et al.* (1987 ▶). For related structures, see: Shan *et al.* (2008 ▶); Fun *et al.* (2008 ▶); Yang (2008 ▶); Ma *et al.* (2008 ▶); Diao *et al.* (2008*a* ▶,*b* ▶); Ejsmont *et al.* (2008 ▶); Qiu & Zhao (2008 ▶).
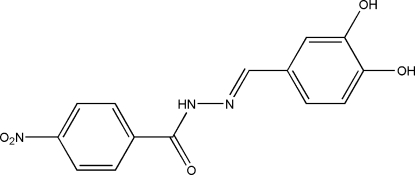


## Experimental

### 

#### Crystal data

C_14_H_11_N_3_O_5_
*M* *_r_* = 301.26
Monoclinic, 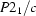

*a* = 7.666 (1) Å
*b* = 13.196 (2) Å
*c* = 13.176 (2) Å
β = 95.361 (3)°
*V* = 1327.1 (3) Å^3^
*Z* = 4
Mo *K*α radiation
μ = 0.12 mm^−1^
*T* = 298 K
0.20 × 0.20 × 0.18 mm

#### Data collection

Bruker SMART CCD area-detector diffractometer
Absorption correction: multi-scan (*SADABS*; Bruker, 2001 ▶) *T*_min_ = 0.977, *T*_max_ = 0.979
8322 measured reflections
3204 independent reflections
1364 reflections with *I* > 2σ(*I*)
*R*_int_ = 0.056

#### Refinement

*R*[*F* ^2^ > 2σ(*F* ^2^)] = 0.069
*wR*(*F* ^2^) = 0.161
*S* = 1.02
3204 reflections
210 parameters
2 restraints
H-atom parameters constrained
Δρ_max_ = 0.17 e Å^−3^
Δρ_min_ = −0.23 e Å^−3^

### 

Data collection: *SMART* (Bruker, 2007 ▶); cell refinement: *SAINT* (Bruker, 2007 ▶); data reduction: *SAINT*; program(s) used to solve structure: *SHELXTL* (Sheldrick, 2008 ▶); program(s) used to refine structure: *SHELXTL*; molecular graphics: *SHELXTL*; software used to prepare material for publication: *SHELXTL*.

## Supplementary Material

Crystal structure: contains datablocks global, I. DOI: 10.1107/S1600536809029705/ci2865sup1.cif

Structure factors: contains datablocks I. DOI: 10.1107/S1600536809029705/ci2865Isup2.hkl

Additional supplementary materials:  crystallographic information; 3D view; checkCIF report

## Figures and Tables

**Table 1:** Hydrogen-bond geometry (Å, °)

*D*—H⋯*A*	*D*—H	H⋯*A*	*D*⋯*A*	*D*—H⋯*A*
O3—H3*A*⋯O2	0.82	2.17	2.636 (4)	116
O2—H2*A*⋯O1^i^	0.82	1.91	2.722 (3)	171
O3′—H3′⋯O1^i^	0.82	1.84	2.548 (7)	144
N2—H2*B*⋯O4^ii^	0.90	2.26	3.121 (3)	158
C5—H5⋯O5^ii^	0.93	2.48	3.210 (4)	135
C10—H10⋯O2^iii^	0.93	2.58	3.467 (3)	159
C11—H11⋯O1^i^	0.93	2.56	3.192 (4)	126

Symmetry codes: (i) 
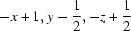
; (ii) 
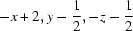
; (iii) 
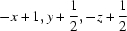
.

**Fig. 1.**
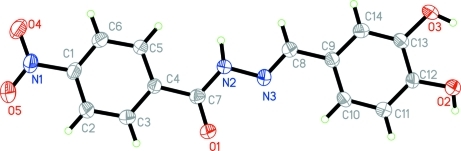
The molecular structure of the title compound with 30% probability displacement ellipsoids for non-H atoms. Only the major disorder component of a hydroxyl group is shown.

**Fig. 2.**
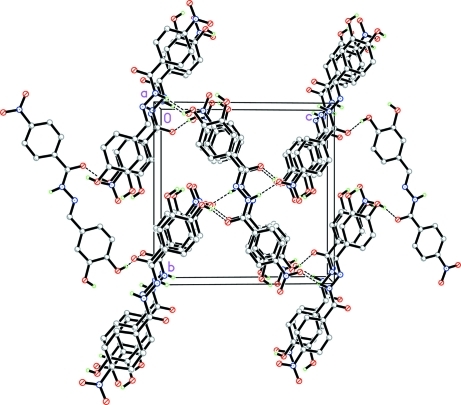
Molecular packing as viewed along the a axis. O—H···O and N—H···O hydrogen bonds are shown as dashed lines.

## supplementary crystallographic information

### Comment

Hydrazones and Schiff bases have been attracted much attention for their excellent biological properties, especially for their potential pharmacological and antitumor properties (Kucukguzel *et al.*, 2006; Khattab *et al.*, 2005; Karthikeyan *et al.*, 2006; Okabe *et al.*, 1993). Recently, a large number of hydrazone derivatives have been prepared and structurally characterized (Shan *et al.*, 2008; Fun *et al.*, 2008; Yang, 2008; Ma *et al.*, 2008; Diao *et al.*, 2008a,b; Ejsmont *et al.*, 2008). As part of the ongoing study (Qiu & Zhao, 2008), we report herein the crystal structure of the title compound.

The molecular structure of the title compound is shown in Fig. 1. The bond distances (Allen *et al.*, 1987) and angles are normal. The dihedral angle between the two benzene rings is 1.6 (1)°. The displays an *E* configuration about the C═N bond. The nitro group is almost coplanar with the attached benzene ring [O4—N1—C1—C6 = -3.5 (5)° and O5—N1—C1—C2 = -3.1 (5)°].

The molecules are linked into layers parallel to the (101) by O—H···O, N—H···O and C—H···O hydrogen bonds (Fig. 2 and Table 1).

### Experimental

3,4-Dihydroxybenzaldehyde (1.0 mmol, 138.1 mg) was dissolved in methanol (50 ml), then 4-nitrobenzohydrazide (1.0 mmol, 181.2 mg) was added slowly into the solution, and the mixture was kept at reflux with continuous stirring for 3 h. After the solution had cooled to room temperature colourless tiny crystals appeared. The tiny crystals were filtered and washed with methanol for three times. Recrystallization from an absolute methanol yielded block-shaped single crystals of the title compound.

### Refinement

One of the hydroxyl groups (O3) is disordered over two distinct sites, with occupancies of 0.643 (5) and 0.357 (5). The C—O distances of the two disorder components were restrained to 1.36 (1) Å. H atoms were placed in calculated positions [O-H = 0.82 Å, N-H = 0.90 Å and C-H = 0.93 Å] and refined using a riding model, with U_iso_(H) = 1.2U_eq_(C) and 1.5U_eq_(O).

### Crystal data

**Table:**

C_14_H_11_N_3_O_5_	*F*(000) = 624
*M**_r_* = 301.26	*D*_x_ = 1.508 Mg m^−^^3^
Monoclinic, *P*2_1_/*c*	Mo *K*α radiation, λ = 0.71073 Å
Hall symbol: -P 2ybc	Cell parameters from 1038 reflections
*a* = 7.666 (1) Å	θ = 2.5–24.5°
*b* = 13.196 (2) Å	µ = 0.12 mm^−^^1^
*c* = 13.176 (2) Å	*T* = 298 K
β = 95.361 (3)°	Block, colourless
*V* = 1327.1 (3) Å^3^	0.20 × 0.20 × 0.18 mm
*Z* = 4	

### Data collection

**Table:**

Bruker SMART CCD area-detector diffractometer	3204 independent reflections
Radiation source: fine-focus sealed tube	1364 reflections with *I* > 2σ(*I*)
graphite	*R*_int_ = 0.056
ω scans	θ_max_ = 28.3°, θ_min_ = 2.2°
Absorption correction: multi-scan (*SADABS*; Bruker, 2001)	*h* = −10→10
*T*_min_ = 0.977, *T*_max_ = 0.979	*k* = −16→17
8322 measured reflections	*l* = −17→10

### Refinement

**Table:**

Refinement on *F*^2^	Primary atom site location: structure-invariant direct methods
Least-squares matrix: full	Secondary atom site location: difference Fourier map
*R*[*F*^2^ > 2σ(*F*^2^)] = 0.069	Hydrogen site location: inferred from neighbouring sites
*wR*(*F*^2^) = 0.161	H-atom parameters constrained
*S* = 1.02	*w* = 1/[σ^2^(*F*_o_^2^) + (0.0491*P*)^2^ + 0.4176*P*] where *P* = (*F*_o_^2^ + 2*F*_c_^2^)/3
3204 reflections	(Δ/σ)_max_ = 0.001
210 parameters	Δρ_max_ = 0.17 e Å^−^^3^
2 restraints	Δρ_min_ = −0.23 e Å^−^^3^

### Special details

**Table:**

Geometry. All esds (except the esd in the dihedral angle between two l.s. planes) are estimated using the full covariance matrix. The cell esds are taken into account individually in the estimation of esds in distances, angles and torsion angles; correlations between esds in cell parameters are only used when they are defined by crystal symmetry. An approximate (isotropic) treatment of cell esds is used for estimating esds involving l.s. planes.
Refinement. Refinement of F^2^ against ALL reflections. The weighted R-factor wR and goodness of fit S are based on F^2^, conventional R-factors R are based on F, with F set to zero for negative F^2^. The threshold expression of F^2^ > 2sigma(F^2^) is used only for calculating R-factors(gt) etc. and is not relevant to the choice of reflections for refinement. R-factors based on F^2^ are statistically about twice as large as those based on F, and R- factors based on ALL data will be even larger.

### Fractional atomic coordinates and isotropic or equivalent isotropic displacement parameters (Å^2^)

**Table:**

	*x*	*y*	*z*	*U*_iso_*/*U*_eq_	Occ. (<1)
O1	0.6818 (3)	1.15446 (15)	0.09056 (17)	0.0606 (6)	
O2	0.5123 (3)	0.56081 (15)	0.27427 (16)	0.0661 (7)	
H2A	0.4520	0.5825	0.3177	0.099*	
O3	0.6235 (5)	0.5128 (2)	0.0971 (3)	0.0814 (15)	0.643 (5)
H3A	0.6013	0.4782	0.1460	0.122*	0.643 (5)
O4	1.0449 (3)	1.43595 (18)	−0.3087 (2)	0.0749 (7)	
O5	0.9668 (4)	1.5401 (2)	−0.1968 (2)	0.1037 (10)	
N1	0.9797 (4)	1.4543 (2)	−0.2301 (2)	0.0648 (8)	
N2	0.7625 (3)	1.03529 (18)	−0.01803 (18)	0.0508 (7)	
H2B	0.7895	1.0090	−0.0778	0.061*	
N3	0.7082 (3)	0.9614 (2)	0.04686 (19)	0.0524 (7)	
C1	0.9174 (4)	1.3697 (2)	−0.1705 (2)	0.0513 (8)	
C2	0.8561 (4)	1.3914 (2)	−0.0783 (3)	0.0601 (9)	
H2	0.8517	1.4579	−0.0552	0.072*	
C3	0.8013 (4)	1.3123 (2)	−0.0209 (2)	0.0572 (9)	
H3	0.7599	1.3255	0.0419	0.069*	
C4	0.8072 (4)	1.2134 (2)	−0.0558 (2)	0.0438 (7)	
C5	0.8690 (4)	1.1948 (2)	−0.1500 (2)	0.0521 (8)	
H5	0.8723	1.1288	−0.1745	0.063*	
C6	0.9252 (4)	1.2736 (2)	−0.2070 (2)	0.0558 (9)	
H6	0.9679	1.2613	−0.2696	0.067*	
C7	0.7446 (4)	1.1325 (2)	0.0104 (2)	0.0475 (8)	
C8	0.7224 (4)	0.8695 (2)	0.0199 (2)	0.0501 (8)	
H8	0.7655	0.8536	−0.0418	0.060*	
C9	0.6702 (4)	0.7893 (2)	0.0869 (2)	0.0468 (8)	
C10	0.6126 (4)	0.8135 (2)	0.1810 (2)	0.0522 (9)	
H10	0.6094	0.8811	0.2008	0.063*	
C11	0.5601 (4)	0.7403 (2)	0.2447 (2)	0.0544 (9)	
H11	0.5232	0.7582	0.3076	0.065*	0.643 (5)
C12	0.5617 (4)	0.6395 (2)	0.2157 (2)	0.0480 (8)	
C13	0.6193 (4)	0.6140 (2)	0.1230 (2)	0.0532 (8)	
H13	0.6224	0.5464	0.1034	0.064*	0.357 (5)
C14	0.6724 (4)	0.6884 (2)	0.0591 (2)	0.0522 (9)	
H14	0.7103	0.6705	−0.0034	0.063*	
O3'	0.5167 (10)	0.7811 (5)	0.3302 (5)	0.076 (3)	0.357 (5)
H3'	0.4225	0.7579	0.3439	0.115*	0.357 (5)

### Atomic displacement parameters (Å^2^)

**Table:**

	*U*^11^	*U*^22^	*U*^33^	*U*^12^	*U*^13^	*U*^23^
O1	0.0805 (16)	0.0506 (14)	0.0549 (14)	0.0031 (12)	0.0283 (12)	−0.0007 (11)
O2	0.0902 (17)	0.0484 (14)	0.0645 (15)	0.0031 (12)	0.0320 (13)	0.0033 (11)
O3	0.140 (4)	0.037 (2)	0.073 (3)	0.005 (2)	0.046 (2)	−0.0088 (18)
O4	0.0773 (17)	0.0780 (18)	0.0740 (18)	−0.0035 (14)	0.0315 (14)	0.0168 (14)
O5	0.155 (3)	0.0525 (17)	0.111 (2)	−0.0188 (18)	0.056 (2)	0.0086 (16)
N1	0.072 (2)	0.052 (2)	0.072 (2)	−0.0078 (16)	0.0208 (17)	0.0113 (17)
N2	0.0662 (18)	0.0403 (16)	0.0484 (16)	−0.0003 (13)	0.0187 (14)	−0.0016 (13)
N3	0.0616 (17)	0.0433 (16)	0.0541 (16)	−0.0017 (13)	0.0149 (14)	0.0068 (13)
C1	0.052 (2)	0.051 (2)	0.053 (2)	0.0002 (16)	0.0145 (17)	0.0112 (16)
C2	0.072 (2)	0.043 (2)	0.068 (2)	0.0001 (17)	0.022 (2)	−0.0005 (18)
C3	0.067 (2)	0.051 (2)	0.056 (2)	0.0041 (18)	0.0228 (18)	−0.0038 (17)
C4	0.0455 (18)	0.0404 (19)	0.0466 (19)	0.0010 (14)	0.0104 (15)	0.0028 (15)
C5	0.066 (2)	0.0407 (19)	0.053 (2)	0.0041 (16)	0.0205 (17)	0.0017 (15)
C6	0.062 (2)	0.056 (2)	0.052 (2)	0.0038 (17)	0.0179 (17)	0.0021 (17)
C7	0.0461 (19)	0.049 (2)	0.048 (2)	0.0023 (15)	0.0087 (16)	−0.0006 (16)
C8	0.054 (2)	0.047 (2)	0.051 (2)	0.0012 (16)	0.0123 (16)	0.0010 (16)
C9	0.0479 (19)	0.0414 (19)	0.052 (2)	−0.0001 (15)	0.0093 (16)	0.0005 (16)
C10	0.064 (2)	0.0330 (18)	0.062 (2)	−0.0018 (15)	0.0148 (18)	−0.0035 (15)
C11	0.066 (2)	0.047 (2)	0.052 (2)	0.0009 (17)	0.0120 (18)	−0.0043 (17)
C12	0.056 (2)	0.0382 (19)	0.052 (2)	−0.0007 (15)	0.0152 (17)	0.0068 (15)
C13	0.062 (2)	0.0387 (19)	0.060 (2)	0.0011 (16)	0.0156 (18)	−0.0049 (17)
C14	0.057 (2)	0.050 (2)	0.052 (2)	0.0006 (16)	0.0160 (17)	−0.0035 (16)
O3'	0.116 (7)	0.066 (5)	0.053 (4)	−0.018 (4)	0.040 (4)	−0.011 (3)

### Geometric parameters (Å, °)

**Table:**

O1—C7	1.236 (3)	C4—C7	1.487 (4)
O2—C12	1.367 (3)	C5—C6	1.375 (4)
O2—H2A	0.82	C5—H5	0.93
O3—C13	1.379 (4)	C6—H6	0.93
O3—H3A	0.82	C8—C9	1.458 (4)
O4—N1	1.216 (3)	C8—H8	0.93
O5—N1	1.221 (3)	C9—C14	1.382 (4)
N1—C1	1.469 (4)	C9—C10	1.390 (4)
N2—C7	1.346 (4)	C10—C11	1.365 (4)
N2—N3	1.386 (3)	C10—H10	0.93
N2—H2B	0.90	C11—O3'	1.319 (5)
N3—C8	1.271 (3)	C11—C12	1.385 (4)
C1—C6	1.360 (4)	C11—H11	0.93
C1—C2	1.373 (4)	C12—C13	1.380 (4)
C2—C3	1.377 (4)	C13—C14	1.379 (4)
C2—H2	0.93	C13—H13	0.93
C3—C4	1.387 (4)	C14—H14	0.93
C3—H3	0.93	O3'—H3'	0.82
C4—C5	1.390 (4)		
			
C12—O2—H2A	109.5	O1—C7—C4	120.4 (3)
C13—O3—H3A	109.5	N2—C7—C4	118.3 (3)
O4—N1—O5	123.0 (3)	N3—C8—C9	119.2 (3)
O4—N1—C1	118.9 (3)	N3—C8—H8	120.4
O5—N1—C1	118.1 (3)	C9—C8—H8	120.4
C7—N2—N3	117.0 (2)	C14—C9—C10	118.1 (3)
C7—N2—H2B	130.3	C14—C9—C8	121.8 (3)
N3—N2—H2B	112.0	C10—C9—C8	120.1 (3)
C8—N3—N2	117.4 (3)	C11—C10—C9	121.5 (3)
C6—C1—C2	122.4 (3)	C11—C10—H10	119.2
C6—C1—N1	119.5 (3)	C9—C10—H10	119.2
C2—C1—N1	118.0 (3)	O3'—C11—C10	110.5 (4)
C1—C2—C3	118.4 (3)	O3'—C11—C12	129.6 (4)
C1—C2—H2	120.8	C10—C11—C12	119.9 (3)
C3—C2—H2	120.8	C10—C11—H11	120.0
C2—C3—C4	120.7 (3)	C12—C11—H11	120.0
C2—C3—H3	119.7	O2—C12—C13	116.3 (3)
C4—C3—H3	119.7	O2—C12—C11	124.3 (3)
C3—C4—C5	119.1 (3)	C13—C12—C11	119.4 (3)
C3—C4—C7	117.4 (3)	C14—C13—O3	121.6 (3)
C5—C4—C7	123.5 (3)	C14—C13—C12	120.3 (3)
C6—C5—C4	120.3 (3)	O3—C13—C12	118.1 (3)
C6—C5—H5	119.9	C14—C13—H13	119.8
C4—C5—H5	119.9	C12—C13—H13	119.8
C1—C6—C5	119.1 (3)	C13—C14—C9	120.8 (3)
C1—C6—H6	120.4	C13—C14—H14	119.6
C5—C6—H6	120.4	C9—C14—H14	119.5
O1—C7—N2	121.3 (3)	C11—O3'—H3'	109.5
			
C7—N2—N3—C8	179.0 (3)	C5—C4—C7—N2	5.9 (4)
O4—N1—C1—C6	−3.5 (5)	N2—N3—C8—C9	178.7 (3)
O5—N1—C1—C6	178.1 (3)	N3—C8—C9—C14	176.0 (3)
O4—N1—C1—C2	175.3 (3)	N3—C8—C9—C10	−2.7 (5)
O5—N1—C1—C2	−3.1 (5)	C14—C9—C10—C11	0.2 (5)
C6—C1—C2—C3	0.2 (5)	C8—C9—C10—C11	178.9 (3)
N1—C1—C2—C3	−178.5 (3)	C9—C10—C11—O3'	178.0 (4)
C1—C2—C3—C4	−0.3 (5)	C9—C10—C11—C12	−0.7 (5)
C2—C3—C4—C5	−0.2 (5)	O3'—C11—C12—O2	1.3 (7)
C2—C3—C4—C7	−179.6 (3)	C10—C11—C12—O2	179.7 (3)
C3—C4—C5—C6	0.7 (5)	O3'—C11—C12—C13	−177.4 (5)
C7—C4—C5—C6	−179.8 (3)	C10—C11—C12—C13	1.1 (5)
C2—C1—C6—C5	0.3 (5)	O2—C12—C13—C14	−179.8 (3)
N1—C1—C6—C5	179.1 (3)	C11—C12—C13—C14	−1.0 (5)
C4—C5—C6—C1	−0.8 (5)	O2—C12—C13—O3	−0.3 (5)
N3—N2—C7—O1	−0.3 (4)	C11—C12—C13—O3	178.5 (3)
N3—N2—C7—C4	178.0 (2)	O3—C13—C14—C9	−178.9 (3)
C3—C4—C7—O1	3.6 (4)	C12—C13—C14—C9	0.5 (5)
C5—C4—C7—O1	−175.8 (3)	C10—C9—C14—C13	−0.1 (5)
C3—C4—C7—N2	−174.6 (3)	C8—C9—C14—C13	−178.8 (3)

### Hydrogen-bond geometry (Å, °)

**Table:**

*D*—H···*A*	*D*—H	H···*A*	*D*···*A*	*D*—H···*A*
O3—H3A···O2	0.82	2.17	2.636 (4)	116
O2—H2A···O1^i^	0.82	1.91	2.722 (3)	171
O3'—H3'···O1^i^	0.82	1.84	2.548 (7)	144
N2—H2B···O4^ii^	0.90	2.26	3.121 (3)	158
C5—H5···O5^ii^	0.93	2.48	3.210 (4)	135
C10—H10···O2^iii^	0.93	2.58	3.467 (3)	159
C11—H11···O1^i^	0.93	2.56	3.192 (4)	126

Symmetry codes: (i) −*x*+1, *y*−1/2, −*z*+1/2; (ii) −*x*+2, *y*−1/2, −*z*−1/2; (iii) −*x*+1, *y*+1/2, −*z*+1/2.
